# Cyclic Olefin Copolymer Microfluidic Devices for Forensic Applications

**DOI:** 10.3390/bios9030085

**Published:** 2019-07-04

**Authors:** Brigitte Bruijns, Andrea Veciana, Roald Tiggelaar, Han Gardeniers

**Affiliations:** 1 Mesoscale Chemical Systems, MESA^+^ Institute for Nanotechnology, University of Twente, Drienerlolaan 5, 7500 AE Enschede, The Netherlands; 2 Life Science, Engineering and Design, Saxion University of Applied Sciences, M. H. Tromplaan 28, 7513 AB Enschede, The Netherlands; 3 NanoLab Cleanroom, MESA^+^ Institute for Nanotechnology, University of Twente, Drienerlolaan 5, 7522 NB Enschede, The Netherlands

**Keywords:** forensic science, microfluidic device, illicit drug analysis, presumptive forensic test, UV-VIS spectroscopy, cyclic olefin copolymer, polymer bonding, polymer surface functionalization

## Abstract

Microfluidic devices offer important benefits for forensic applications, in particular for fast tests at a crime scene. A large portion of forensic applications require microfluidic chip material to show compatibility with biochemical reactions (such as amplification reactions), and to have high transparency in the visible region and high chemical resistance. Also, preferably, manufacturing should be simple. The characteristic properties of cyclic olefin copolymer (COC) fulfills these requirements and offers new opportunities for the development of new forensic tests. In this work, the versatility of COC as material for lab-on-a-chip (LOC) systems in forensic applications has been explored by realizing two proof-of-principle devices. Chemical resistance and optical transparency were investigated for the development of an on-chip presumptive color test to indicate the presence of an illicit substance through applying absorption spectroscopy. Furthermore, the compatibility of COC with a DNA amplification reaction was verified by performing an on-chip multiple displacement amplification (MDA) reaction.

## 1. Introduction

Lab-on-a-chip (LOC) technology can be used to decrease analysis time, the amount of reagents and analyte, and can add to the development of portable devices that can be used directly on the crime scene. An LOC comprises an enclosed microfluidic channel network with small characteristic internal dimensions, typically below 1 mm, in which reagents can be manipulated on the microscale. Because of sample handling in a sealed microfluidic environment, LOC systems reduce the risk of (cross-) contamination and can be designed for single use—with a unique barcode for easy tracking—two beneficial attributes that help to improve the chain of custody.

The material choice for microfluidic devices is important. Frequently used LOC materials, such as silicon, glass, and polydimethylsiloxane (PDMS), have their own characteristics in terms of transparency, chemical resistance, and biochemical compatibility. Silicon is widely used as chip material because of its good thermal conductivity, which makes it suitable for the fast heating and cooling required in (well-based) polymerase chain reaction (PCR) cycling [1,2,3]. However, it is not transparent for UV-VIS light. Because of its transparency in the visible range and, therefore, the possibility for optical detection, glass is widely used as chip material for (forensic) DNA analysis on-chip. In order to minimize the absorption of the amplification mixture on glass, the material must be coated [4,5]. PDMS is biochemically compatible, transparent, and easily moldable, but is not compatible with common solvents [6,7,8,9]. 

In general, plastics are attractive because of their biocompatibility [10]. Therefore, microfluidic devices for biological fluid analysis can also be made from plastics other than PDMS, such as, cyclic olefin copolymer (COC) [11,12,13,14] or poly(methyl methacrylate) (PMMA) [15,16,17,18]. Other novel and interesting materials (for forensic chips) could be SU-8 [19,20], NOA81 [21,22], or poly(ethylene glycol) diacrylate (PEGDA) [23,24].

COC is widely known for food packaging and medical/diagnostic disposables. Characteristics of COC include its low water absorptivity (<0.01%) and good electrical insulating properties [11,12,25]. Long-term stability of surface treatments have been reported, and the material is resistant to acids (e.g., HCl (hydrogen chloride), H_2_SO_4_ (sulphuric acid), and HNO_3_ (nitric acid)), alkalines (e.g., NaOH (sodium hydroxide) and NH_4_OH (ammonia solution)), and polar solvents (e.g., C_2_H_6_O (ethanol) and C_3_H_6_O (acetone)). Only non-polar organic solvents, such as toluene or hexane, attack the material [1]. Other characteristics include its high rigidity and high optical transparency, even within the UV range. Depending on the grade of the COC, the heat deflection temperature (the temperature at which a polymer deforms under a specified load) varies between 70 °C and 170 °C [13,25].

Various fabrication techniques are available to create microfluidic networks in COC, such as micromilling, hot-embossing, and injection molding [11,12]. Micromilling, the technique used in this study, is a fabrication method that can be described as the creation of microscale features via rotating cutting tools (e.g., mills and drills) that remove bulk material. By conversion of a three-dimensional model (e.g., drawn in SolidWorks) to a computer-aided design, which can be imported into a computer numerical control (CNC) machine, it is easy and fast to convert a design to a prototype, without the need of a mold. The precision and resolution of CNC milling are currently in the microscale range. The surface roughness of COC after milling is in the range of 0.4–2 µm [26]. 

Two (processed) plates of COC can be bonded together by various methods, including gluing, thermal bonding, or solvent bonding [11,12]. A suitable solvent for solvent bonding, providing a strong bond, has a Hildebrand parameter (defined as the square root of the cohesive energy density) close to the parameter of the material to be bonded. For COC, two solvents with a Hildebrand parameter close to that of COC (δ = 17.7 (J/cm^3^)^1/2^ are cyclohexane (δ = 16.7 (J/cm^3^)^1/2^ and acetone (δ = 20.4 (J/cm^3^)^1/2^ [4]. An alternative method is the sealing of microfluidic devices by the use of tape that is biocompatible. Commercially available PCR adhesive tape, ThermalSealRTS (Excel Scientific), can withstand a pressure of more than 3 bar upon the sealing of a straight channel (200 µm in width and depth, 15 mm in length) in COC. Serra et al. used such a seal for a COC chip containing a straight channel (300 µm in width and depth) and a middle circular chamber (6 mm diameter) to carry out a PCR reaction. The seal could withstand high temperature cycling, while the reagents were in direct contact with the tape [27].

Several microfluidic procedures have been reported for the analysis of drugs of abuse, mainly based on the presence of illicit substances within body fluids and/or analysis by capillary electrophoresis [28,29,30,31,32,33]. Musile et al. made a paper microfluidic device in which the unknown sample is split up into six different lanes, which can detect different types of compounds based on conventional color tests [34]. This paper microfluidic device was further developed to detect seized cocaine samples by the use of gold nanoparticles, resulting in a color change upon the aggregation of the nanoparticles with cocaine [35]. Krauss et al. developed a centrifugal microdevice for the detection of cocaine (cobalt thiocyanate test) and methamphetamine (Simon’s reagent) with smartphone analysis. Probably due to the relatively short optical path length of only 284 µm, the difference between the hue values of the positive and negative control was not discriminative enough. By also using the saturation value, the obtained threshold values for the detection of cocaine and methamphetamine were 0.25 mg/mL and 0.75 mg/mL, respectively [36].

Multiple displacement amplification (MDA) is an isothermal amplification method, which can be completely carried out at 30 °C. This whole genome amplification method makes use of random hexamer primers and phi29 DNA polymerase [37]. After 1.5–2.0 h, 4–7 µg of DNA will be generated in a volume of 20 µL (i.e., 200–350 ng/µL) upon starting with 10 ng of purified DNA [38]. MDA can be used as a technique to amplify the DNA prior to further forensic analysis. Sufficient DNA for sequencing by MDA from a single cell can be achieved [39]. Ballantyne et al. showed that it is possible to use MDA to amplify genomic DNA from small amounts of template, 5 pg to 1 ng, for downstream short tandem repeat (STR) multiplex genotyping [40]. They also showed that by using MDA, the quality and quantity of DNA can be increased and that it has the potential to improve STR-typing from difficult samples in forensic casework [41]. Bienvenue et al. designed a chip for forensic DNA analysis via PCR amplification of STR fragments. A conventional thermocycler was used for the amplification reaction on-chip. Detection and analysis were carried out off-chip, with conventional capillary electrophoresis (CE) [42]. A 9-plex STR profile from 2.5 ng input standard DNA in about 3 h was acquired on-chip by Liu et al. [43]. Xu et al. have developed a PCR chip for a forensic test. With this, the disposable device short and long DNA fragments could be amplified, as well as STRs. Norland Optical Adhesive (NOA) 81 was used as chip material. The STR analysis was carried out off-chip with a conventional CE system [44].

Two forensically relevant reactions within COC chips were studied in this research, namely the spot test for illicit drug analysis and DNA amplification. First, the use of COC chips in a presumptive test for illicit drugs was investigated. To acquire more information about a suspicious powder, a color or spot test is commonly carried out by airport security or the police. These color tests are simple, quick, and relatively sensitive. The best results can be acquired with sample quantities of less than one milligram or with a pure substance, and the result can be observed with the unaided human eye [45,46,47]. However, the latter property also brings the main drawback of these tests—subjective interpretation. Although these tests are selective, they are not very specific. Only a certain class of compounds (e.g., secondary amines, such as methamphetamine) can be identified [36,45,48]. Since these tests are presumptive, additional confirmatory analysis by an accredited forensic laboratory has to be carried out [49]. Moreover, the used hazardous and corrosive chemicals are present in a semi-open environment. By performing these presumptive tests in a chip that can be easily combined with a (handheld) UV-VIS spectrometer, more analytical information can be obtained from the analytes in a manner that is safe for the investigator. Since the exact wavelength of absorption can be determined, the additional information about the compound and subjective observation by the unaided human eye is excluded. The suspicious compound can, subsequently, be confirmed by the forensic laboratory with a test for exact identification, or the obtained spectrum may already directly be compared with spectra in a database.

Secondly, the compatibility of COC chips with biochemical reactions is explored by performing a multiple displacement amplification (MDA) reaction in microfluidic chips with and without a bovine serum albumin (BSA) coating. The biochemical compatibility of chip material is of great importance in several applications in forensics, where DNA amplification, which is also investigated in this research, is perhaps the one that is best known. The interaction of the used amplification mixture with the surface can result in a delay of the reaction, or even total inhibition, as is the case, for example, with glass, silicon, and PDMS surfaces [50,51,52,53]. This can be avoided by passivation of the walls of the reaction chamber with a coating prior to use [50]. Bovine serum albumin (BSA) is widely used to coat the channel walls in order to prevent absorption of the polymerase [50,54].

## 2. Materials and Methods

### 2.1. Chemicals

All the used chemicals were obtained from Sigma-Aldrich (Zwijndrecht, The Netherlands) unless stated otherwise.

### 2.2. COC Chips

#### 2.2.1. Chip Fabrication

The chip designs for the color test chip and the MDA chip were made by means of SolidWorks software. The channels were milled in an 85 mm × 54 mm × 2 mm COC plate (DENZ Bio-Medical grade 6013, Götzig, Austria) using a Sherline 5410 Deluxe Mill with a 1 mm diameter mill. Both the inlet and outlet were drilled with a 1.5 mm diameter drill. The in- and outlet of the COC chips had a diameter of 1.5 mm in order to insert the standard pipette tips directly. Therefore, the chips could be easily filled by the use of a pipette, without additional tubing or chipholder with fluidic connections. After milling, the chips were thoroughly cleaned with MilliQ water, ethanol, and isopropanol, and finally blown dry with nitrogen gas. To regain optical transparency after milling, by decreasing the surface roughness as suggested by Ogilvie et al. [55], the chips were exposed to cyclohexane vapor of 60 °C for 1 min. Note that because of this manual process, the transparency might differ slightly from chip to chip, and, therefore, calibration of each individual chip was required.

#### 2.2.2. Design of the Presumptive Color Test Chips

**Figure 1:**
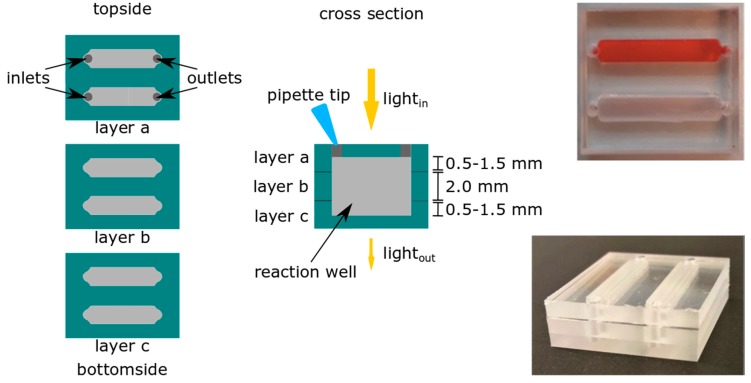
Schematic representation of cyclic olefin copolymer (COC) chips for the color test with an optical path length of 2, 3, or 4 mm for integrated UV-VIS spectroscopy. The insets show chips with an optical path length of 4 mm. The top channel in the top right picture is filled with Allura Red.

The design of the color test chip consisted of two channels, as depicted in Figure 1, which can be utilized to obtain a reference spectrum in channel 1 (pure reagent) and a spectrum of the analyte solution in channel 2 (reagent and analyte), or for simultaneous testing of different reagents. Channel dimensions were 15 mm and 3 mm in length and width, respectively. The channel height in each COC layer depended on the milling depth, and was 0.5, 1, 1.5, or 2 (completely through) mm, thereby obtaining optical path lengths of 2, 3, or 4 mm, as can be seen in Figure 1 and Table 1.

**Figure 2:**
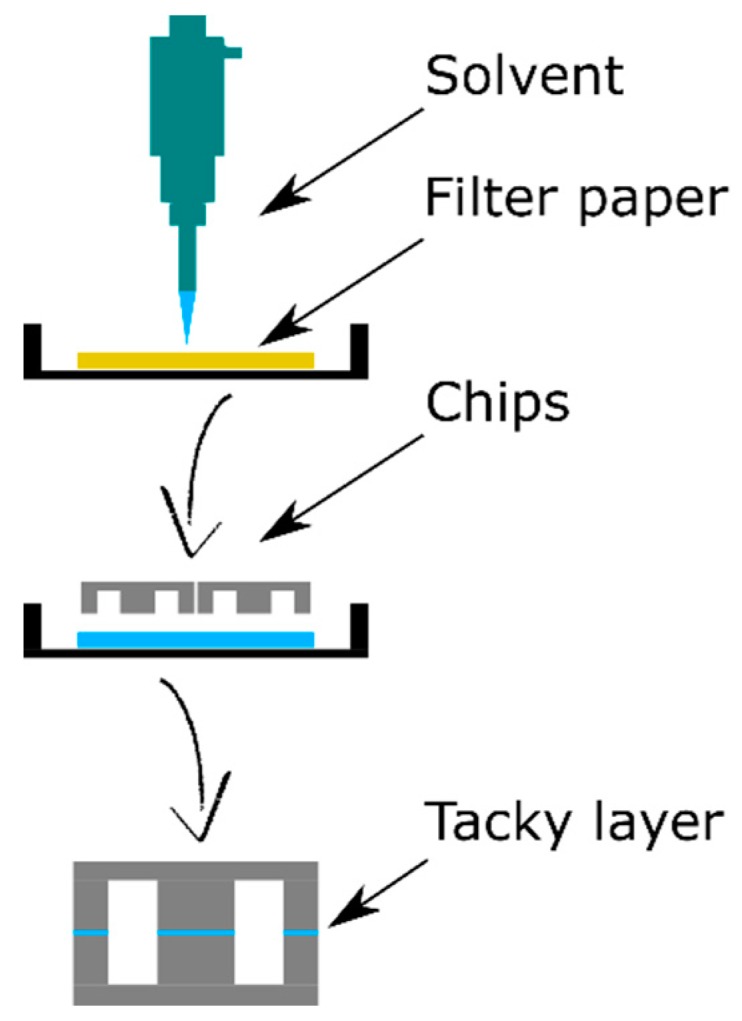
Schematic side-view of the COC bonding process using the tacky layer method. It starts by adding a piece of filter paper to a Petri dish and soaking it with a mixture of acetone and cyclohexane. The chip parts were placed on top of the filter paper for 2 min, subsequently aligned, and a 2 kg weight was placed on top for 5 min.

The COC layers for the drug chips were bonded by creating a tacky COC layer, a solvent bonding method suggested by Keller et al. [56], which is shown in Figure 2. Each COC part was exposed for about 2 min to a mixture of 40 vol% cyclohexane and 60 vol% acetone by adding this mixture onto a piece of filter paper (Whatman Hardened, Ashless, Grade 541 Filtration Paper). Subsequently, the parts were manually aligned, and a 2 kg weight was placed on top for 5 min at room temperature.

#### 2.2.3. Design of the MDA Chips

**Figure 3:**
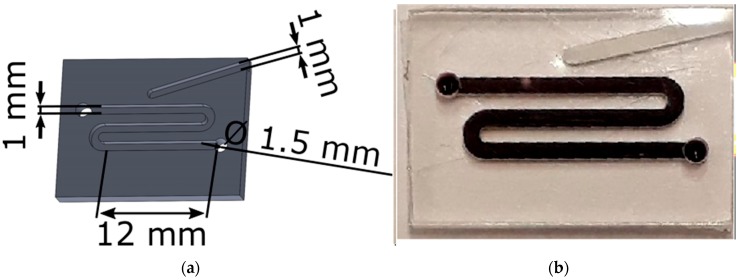
Multiple displacement amplification (MDA) chips with (**a**) the schematic representation of the MDA chips with the used dimensions and (**b**) a chip filled with food dye to show the channel design.

The COC chip for MDA had a meandering channel with both a width and a depth of 1 mm. On the top, a dead-end channel was included, which was used to insert a thermocouple close to the reaction channel for temperature control. The other dimensions of the chip can be seen in Figure 3a. In Figure 3b, the chip was filled with food dye to show the channel design.

The channels in the chips were sealed with Microseal ‘B’ Adhesive Seals (Bio-Rad, Veenendaal, The Netherlands). Also, the in- and outlet were sealed with this tape after the filling of the chip with the MDA mixture.

### 2.3. Presumptive Color Test

#### 2.3.1. Reagents and Analytes

For the Marquis reagent, 50 mL of 96% sulphuric acid was added to 5 mL of 36% formaldehyde.

In order to test UV-VIS absorption quantitively in the chips, Allura Red (E129) food dye was used.

Since our lab does not have the license needed to work with drugs of abuse (e.g., amphetamine, methamphetamine, or cocaine), as a proof of principle of qualitative analysis, drug analogues were used. Acetylsalicylic acid, as well as lidocaine—both known to give a false positive with the Marquis reagent—were directly dissolved in the reagent. Benzodioxolyl-N-methylbutanamine (MBDB) was kindly gifted by the Netherlands’ Forensic Institute, and was used as a closely related analogue of 3,4-methylenedioxymethamphetamine (MDMA) to demonstrate the proof-of-principle.

#### 2.3.2. UV-VIS Spectroscopy

On-chip spectra were recorded with a Maya2000 Pro spectrometer and a HPX2000 lamp (both Ocean Optics Inc., Duiven, The Netherlands). LabVIEW (National Instruments, Austin, TX, USA) was used to record and analyze the spectra in the wavelength region of 350 to 750 nm (visible range). In order to obtain control spectra off-chip, the same set-up was used in combination with a quartz suprasil precision cuvette (Hellma Analytics, Müllheim, Germany).

### 2.4. MDA

#### 2.4.1. MDA Protocol

MDA reactions were carried out with the illustra GenomiPhi V2 DNA Amplification Kit (GE Healthcare, via Fisher Scientific, Landsmeer, The Netherlands). MDA mix was made with reaction buffer, sample buffer, and enzyme mix, TaqMan Control Genomic DNA (human, male, 10 ng/µL) (Applied Biosystems, via Fisher Scientific, Landsmeer, The Netherlands), and MilliQ in a volumetric ratio of 9:5:1:1:4.

From the mix, 10 µL was injected in the chip and subsequently the in- and outlet were sealed. The chip was placed in a water bath of 32 °C for 2 h. Prior to MDA mixture injection, some chips were coated with a 1% BSA solution. Also, 10 µL MDA mixture was pipetted into a 500 µL Eppendorf polypropylene vial as a control.

Another control with a volume of 5 µL was carried out with a conventional thermocycler. The amplification reactions were carried out in hard-shell full-height 96-well semi-skirted PCR plates with a pierceable PCR plate heat seal foil (both from Bio-Rad). To carry out the amplification protocol, a CFX386 real-time PCR thermal cycler with associated software from Bio-Rad was used.

#### 2.4.2. DNA Quantification

The concentration of double-stranded DNA (dsDNA) was determined with the Qubit dsDNA HS Assay Kit and the fluorescence was measured using the Qubit 3.0 fluorometer (both from Thermo Fischer Scientific, Landsmeer, The Netherlands). All samples were measured in triplicate.

## 3. Results and Discussion

### 3.1. Presumptive Color Test Chips

#### 3.1.1. Food Dyes

**Figure 4:**
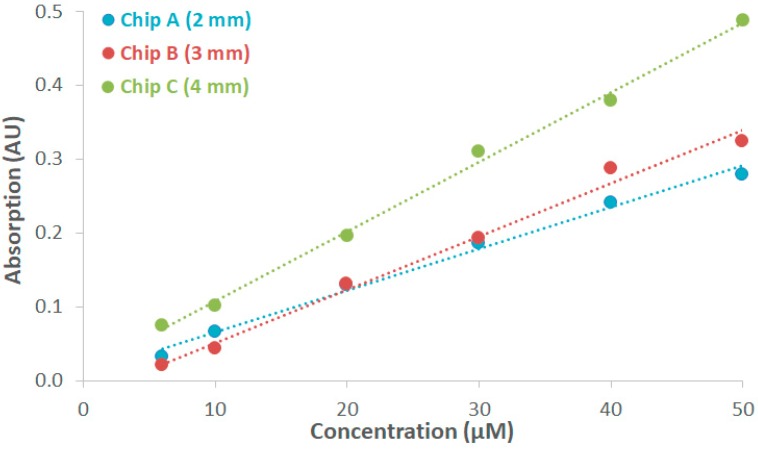
Absorption measurement curves of the absorption of Allura Red in chips A, B, and C versus the concentration at a wavelength of 504 nm.

The absorption of Allura Red was measured quantitively in COC chips with optical path lengths of 2, 3, and 4 mm, respectively. The maximum measurement on-chip and off-chip (cuvette setup) was present at 504 nm, which was in accordance with the information from the supplier [57]. A 0.2 mM stock solution of Allura Red was prepared, and a dilution series from this stock solution was prepared to obtain concentrations of 6 µM, 10 µM, 20 µM, 30 µM, 40 µM, and 50 µM. Figure 4 depicts the linear relationship between the absorption value at 504 nm and the varied concentration of Allura Red. A comparison was made between chips A, B, and C, and it was found that increasing the optical path length resulted in a higher absorption value. The molar absorptivity was determined for both the on-chip and off-chip measurements. With the cuvette, a molar absorptivity of 22,047 M^−1^cm^−1^ was calculated, which is in accordance with the specifications of the supplier (≥20,000 M^−1^cm^−1^) [57]. For chips A, B, and C, values of 28,835 M^−1^cm^−1^, 21,406 M^−1^cm^−1^, and 23,845 M^−1^cm^−1^ were obtained, respectively. Note that the transparency of the chips is not 100% because of some imperfections in the COC, and, as a consequence, the obtained on-chip values for the molar absorptivity vary slightly. This is also the reason why the absorption measurement curves did not cross the origin (0,0).

#### 3.1.2. Drug Analogues

**Figure 5:**
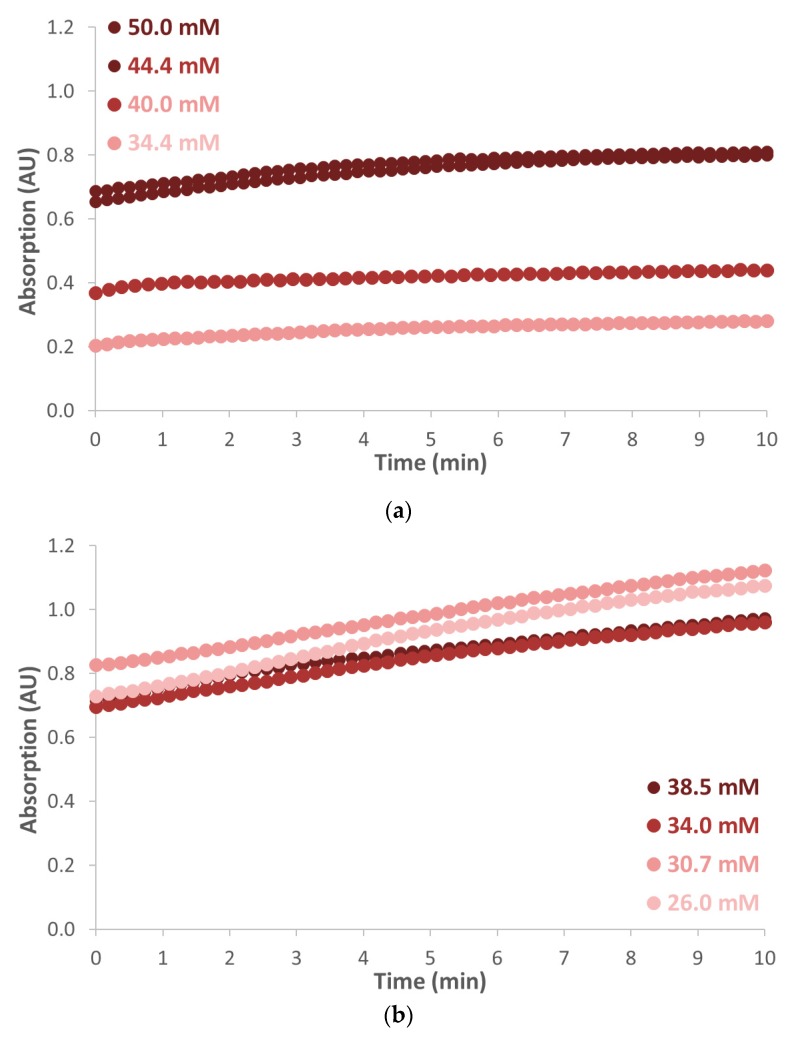
Absorbance measured at 500 nm for (**a**) acetylsalicylic acid dissolved in the Marquis reagent measured in COC chip D and (**b**) lidocaine dissolved in the Marquis reagent measured in COC chip A.

Both acetylsalicylic acid and lidocaine were tested for qualitative analysis with the Marquis reagent in chips D and A, respectively. Pure Marquis reagent was used as blank. Since it is known that the color intensity of lidocaine and acetylsalicylic acid dissolved in Marquis reagent varies over time, samples with different concentrations were prepared. Afterwards, the acetylsalicylic acid powder was mixed with the Marquis reagent and it was added to chip D with a pipette. The measurement started exactly 7 min after the mixing of the compounds, and the absorption was measured for a duration of 10 min. The spectrum gave a clear increasing peak around 500 nm, which was in accordance with the visible red color. The absorbance measured for acetylsalicylic acid dissolved in the Marquis reagent in a concentration range of 34.4–50.0 mM, which is shown in Figure 5a. Over time the color became more intense, which was also observed by the unaided human eye. For lidocaine dissolved in the Marquis reagent, a concentration range of 26.5–38.5 mM was used in combination with chip A. Also, for lidocaine and Marquis, a visible red color was observed in combination with a peak in the absorbance spectrum around 500 nm. Again, the intensity of the red color increased over time for all concentrations, as can be seen in Figure 5b. Since both analytes show an absorption maximum at the same wavelength, it was not possible to distinguish between these two analytes by absorption spectroscopy. Note that in both cases, the color intensity was not proportional to the concentration of the drug substitutes, which might be related to the rather complex chemistry of the Marquis test [58]. We did not investigate this any further.

**Figure 6:**
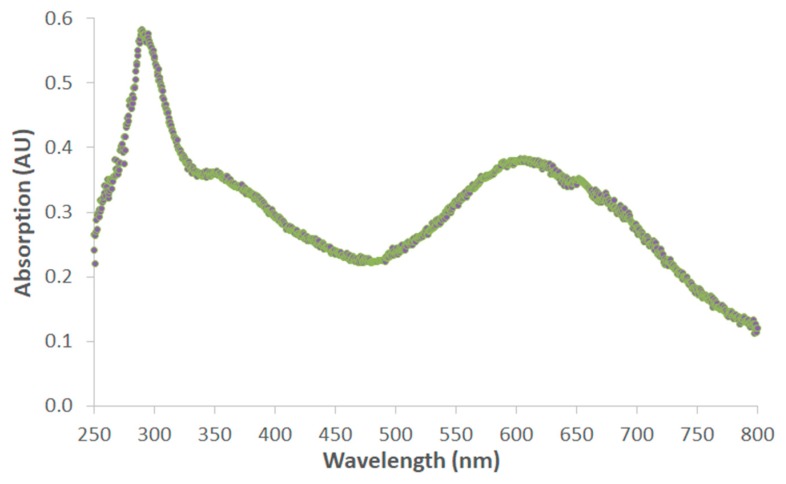
Absorption spectrum of benzodioxolyl-N-methylbutanamine (MBDB) dissolved in Marquis reagent with a concentration of 50 mM.

In order to verify the applicability of COC chips for absorption spectroscopy for suspicious compounds, the absorption curve of MBDB was also measured. MBDB was directly dissolved in the Marquis reagent with a concentration of 50 mM MBDB. The Marquis showed a dark blue to black (black-ish purple) color upon the addition of MBDB. Figure 6 shows the absorption spectrum of Marquis with MBDB, with peaks around 600 nm (broad) and 300 nm (sharp), which correspond to a blue/purple color and absorption in the ultraviolet region, respectively.

### 3.2. MDA Chips

**Figure 7:**
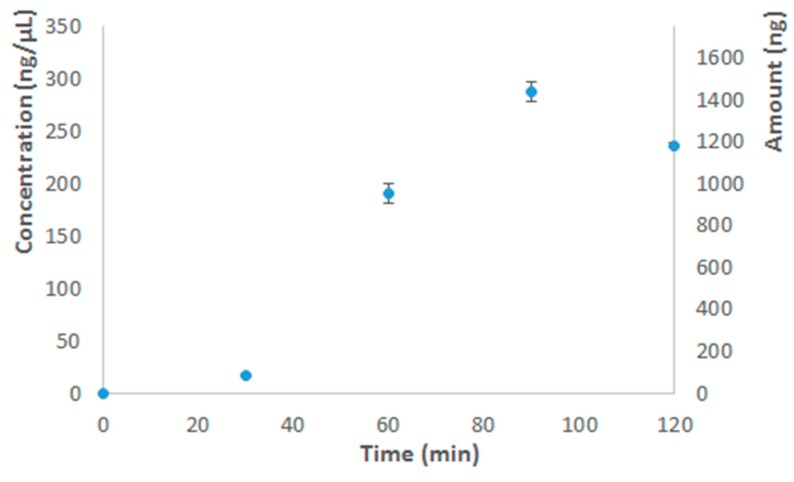
The amplification yield expressed in both concentration (ng/µL) and amount of dsDNA (ng) versus the amplification time with an input of 2.5 ng of purified DNA (TaqMan Control Genomic DNA). The error bars are ± 1 standard deviation.

The results of the DNA quantification are shown in Table 2. After a 2 h run, the average DNA concentration for uncoated chips was well above 200 ng/µL, which was in accordance with the run in the thermocycler (Figure 7) and the literature [37,38,59]. For purified DNA, the recommended amplification time by the manufacturer is 1.5 h, since the exonuclease activity of the polymerase enzyme might otherwise start to degrade the amplification product, which explains why after 2 h less DNA was measured than after 1.5 h (Figure 7). Since the MDA reaction could be successfully performed in the uncoated COC chips, this was further explored, which is described in detail elsewhere [60].

### 3.3. Additional Remarks and Future Perspectives

#### 3.3.1. Drug Chips

Absorption spectra from a food dye, as well as from two drug analogues, could be obtained. This demonstrates the proof-of-principle of the use of COC as a suitable chip material when high optical transparency is required. In order to fully demonstrate the use of a microfluidic device for illicit drug testing, field tests with actual suspicious compound(s) are required. A first step was made by using MBDB, a closely related analogue of MDMA, and with similar effects as MDMA.

It must be noted that the color intensity of the product (of the reagent and the analyte) changed over time (as also can be seen in Figure 5), which made the interpretation of quantitative measurements challenging; however, for a presumptive test, this is not of relevance, as additional (quantitative) testing by a forensic laboratory is still required to identify the suspicious compound(s).

Keller et al. investigated the bond strength of the tacky layer method and observed no leakage for the COC–COC bond up to a pressure of 744 kPa. In fact, no leakage was observed during the experiments in this work for the drug chips bonded with this method.

Future perspectives include the involvement of on-chip storage of the reagents and smartphone-based detection with data storage to be directly applicable in the field and further increase the chain of custody. LOC devices are also very suitable for multiplex reactions, which allow the performance of multiple color tests with only one sample of the suspicious compound.

#### 3.3.2. MDA Chips

It is remarkable that, although the coating of a COC chip with BSA has been described in the literature to prevent non-specific binding to a COC surface in biological applications (e.g., PCR or immunosensors) [7,54,61], in our experiments, a BSA coating clearly resulted in an inhibitory effect on the MDA reaction. A reason for this could be that, as has been reported by Zhang et al., salts present in amplification mixtures (e.g., KCl, NaCl, MgSO_4_, or MgCl_2_) might affect the coating, and, hence, BSA could interact with the fluorescent dye.

MDA can be used to amplify the DNA prior to PCR. To further investigate the quality of the DNA after MDA amplification on-chip, methods such as STR analysis can be performed.

## 4. Conclusions

In COC chips, a linear relation between the absorption of the Allura Red food dye as a function of the dye concentration was measured. In addition, with acetylsalicylic acid and lidocaine, both dissolved in the Marquis reagent, and reliable and reproducible absorbance spectra could be obtained, as the chips showed sufficient optical transparency in combination with chemical resistance to strong acids. Therefore, a subjective observation by means of the unaided human eye can be avoided by using these COC chips in combination with absorbance measurements.

The biochemical compatibility of COC concerning an isothermal amplification reaction is very good. Coating of the channel walls with BSA gave lower amplification yields than without any coating in combination with the MDA reaction. With uncoated chips, amplification yields could be obtained that are in accordance with data generated with a conventional thermocycler and with the yields reported by the manufacturer as well as in the literature.

Milling of COC chips is suitable for fast prototyping. Depending on the application, either solvent bonding or sealing with tape can be used, which are both fast and simple techniques. Solvent bonding is suitable when two pieces of COC have to be bonded together. For closing the channels in a biocompatible manner, sealing is the method of choice. This makes COC a chip material suitable for various applications (e.g., forensic or medical) that demand microfluidic chips with high optical transparency, high chemical resistance, and/or good compatibility with biochemical reactions. The ease of fabrication and good characteristics of COC also allow the possibility of multiplex analysis (i.e., performing several reactions at the same time). For instance, one suspicious compound can be tested in one run with different color tests to obtain more information at once. Nevertheless, a variety of additional experiments with COC chips is required before a decision can be made as to whether COC chips are applicable as an alternative for well-known and well-embedded forensic applications.

## Figures and Tables

**Table 1:** Milling depth in each COC layer and optical path length for various chip designs.

Chip	Layer a (mm)	Layer b (mm)	Layer c (mm)	Optical Path Length
A	1.0	-	1.0	2.0
B	0.5	2.0	0.5	3.0
C	1.0	2.0	1.0	4.0
D	1.5	-	1.5	3.0

**Table 2:** The concentration of double-stranded DNA (dsDNA) obtained after 2 h of amplification with the Eppendorf vials and chips with and without bovine serum albumin (BSA) coating (n = 3).

	Run 1 (ng/µL)	Run 2 (ng/µL)	Run 3 (ng/µL)	Run 4 (ng/µL)	Average ^1^ (ng/µL)
Eppendorf vial	204 ± 7	218 ± 8	265 ± 13	200 ± 16	222 ± 30
Chip without BSA coating	118 ± 6	209 ± 6	295 ± 13	233 ± 20	214 ± 73
Chip with BSA coating	79 ± 3	^2^	86 ± 2	62 ± 3	76 ± 12

^1^ The standard deviation was taken from the average values of the four runs. ^2^ The seal of the chip came off, hence no quantification measurement could be carried out.
